# Book Review

**Published:** 1929

**Authors:** 


### Reviews of Books

**Medical Essays.** By C. O. Hawthorne, M.D. Pp. iv., 246. London: John Bale, Sons & Danielsson Ltd. 1928. Price 4s. 6d.—This book contains 49 essays upon subjects which are peculiar to the training and practice of the medical profession. In the opening chapters, dealing with education, the author claims that the width of the training entailed constitutes one of the most attractive features of the medical profession, but he regards the curriculum as being by no means beyond criticism, and insists upon the great importance of clinical as compared with academic teaching. He considers that lectures are essential because "the inspiration of the living teacher is as necessary in medical training as are hospital practice and library study." In discussing the therapeutic value of drugs the author maintains that they should not be neglected simply because they may be powerless to neutralize directly the cause of disease; for, on the other hand, they may assist nature by relieving untoward symptoms. In a collection of useful and interesting essays, such as this book presents, it would be impossible to select the most important, but the short chapter dealing with professional secrecy must commend itself to all who read it. Each essay is readable, well put, and nicely expressed.

**Medical Adventure.** By E. Ward, M.D., F.R.C.S. Pp. xii., 291. London: John Bale, Sons & Danielsson Ltd. 1929. Price 8s. 6d.—We have not met a book quite like this before. It is a series of essays strung together by no link other than that of the author's personality. We confess that we took it up for review with no expectation of enjoying it, but the first chapter—that on legal adventures—was so alluring that we went straight on with it, and found much that was entertaining, much that was instructive, and almost nothing that was dull. Some of the clinical essays—for example, that on erythema nodosum, the conclusion of which agrees closely with that of Dr. Symes's Long Fox Lecture—prove that Dr. Ward is not merely a clever and attractive writer but also a careful observer. Finally, he may be congratulated on being in a position to write that last essay, one entitled, "A subjective study of encephalitis lethargica." Articles by doctors on their own illnesses are always very instructive. Altogether this book is excellent value for the money.

**Mirror Writing.** By MACDONALD CRITCHLEY, M.D., M.R.C.P. Psyche Miniatures, Medical Series No. 11. Pp. 80. London: Kegan, Paul, Trench, Trübner & Co. 1928. Price 2s. 6d.—In this brief "libel" (to use the term in its original sense) Dr. Critchley gives an admirable account of the curious phenomena of "mirror writing," "inverted writing" and "backward speech." He describes how these phenomena may occasionally be the accompaniments of subnormal mental powers, but he quite fairly quotes the instance of the best known of all mirror writers, Leonardo da Vinci, who wrote mirrorwise from the age of twenty, whose mental faculties, although amounting to genius, were never in the least "to madness near allied." On the whole Critchley perhaps favours the theory of mere left-handedness as best explaining "mirror writing," though he recognizes the difficulty of accepting this to explain why Hebrew, Hindustani and Arabic writing should run from right to left. He adds an interesting note of a personal inquiry which he made, showing that out of 107 Egyptian clerks at the Egyptian Ministry of Health only one could write more easily with the left hand than the right. But it is an entertaining account of a series of little understood speech—and writing—diversities. |

**Cytoarchitectonics of the Human Cerebral Cortex.** By C. VON ECHONOMO. Translated by DR. S. PARKER. Pp. xii., 186. Illustrated. London: Oxford University Press. 1929. Price 21s.—This book, as its title implies, deals with the detailed cellular structure of the cerebral cortex. In recent times, with advances in the study of psychiatry and mental deficiency, more and more attention is centring around the detailed structure of the cerebral cortex, and considerable progress is attending efforts to correlate structure with function. Von Echonomo has expended a vast amount of time and labour in determining the differences in minute structure attaching to the various regions of the grey matter of the cerebral cortex. It is, perhaps, a little depressing to the average reader to realize that he has succeeded in differentiating no less than 109 areas of varying structure. However, certain fundamental groupings stand out from the general mass of detail. The rhinencephalon, which occupies but a tiny portion of the human cortex, appears essentially different in its histology from all the rest of the cortex, while the general six-layer lamination is recognizable over the rest of the grey matter of the cerebrum. The relative development of the various layers differs widely—the broad distinction being between the relative preponderance of pyramidal and of granular cells—pyramidation being significant of efferent functions, and granulation of cortical receptive areas. One of the difficulties for the reader is the extensive new terminology which is used throughout the book, and it is difficult, in the main, to harmonize most of the author's descriptions with those appearing in older text-books. This difficulty, however, appears inevitable where a large amount of new knowledge is added to our previous store. In the last chapter the general implications which follow from the detailed structure of the cerebral cortex are well summarized, and present a comprehensive survey of the book, which should prove very readable to anyone who is interested in general conclusions but finds detailed histology irksome. The book is beautifully illustrated with a wealth of excellent microphotographs which are described in so much detail that they require very close attention. The book is undoubtedly a valuable addition to our knowledge of the branch of neurology, which is only beginning to receive the attention it deserves.

**Low Blood Pressure, its Causes and Significance.** By J. F. HALLS-DALLY, M.A., M.D., M.R.C.P. Pp. xvii., 257. London: William Heinemann Ltd. 1928. Price 15s. —Dr. Halls-Dally follows up his book on *High Blood Pressure* with a companion volume dealing with the other extreme. He says truly, "Although the literature of medicine abounds with references to high blood pressure, little mention is made of the conditions under which low blood pressures occur." So this volume is devoted to a consideration of all the conditions with which low arterial pressure may be associated. Briefly they may be grouped as acute infections, chronic infections, chronic wasting diseases, endocrine defects and the little understood conditions of shock and collapse. The author rightly attributes to "hypopiesis" many of the symptoms met with in neurasthenia, "the subject is more often than not apathetic, miserable and distressed." He points out how small a rise in the systolic pressure may be attended by marked benefit in the feelings and state of the victim. One symptom on which he lays stress is the insomnia of hypopiesis, which, he says, "occurs early in the morning, the patient getting off to sleep without much difficulty, but becoming wakeful from about 2 a.m. onwards, with snatches of broken sleep, from which he wakes weary and unrefreshed." Altogether this is a book full of careful and practical observations, with many valuable hints on treatment.

**Nephritis.** By A. C. ALPORT, M.D., M.R.C.P. Pp. xvi., 175. Illustrated. London: Wm. Heinemann Ltd. 1929. Price 7s. 6d.—The author is to be congratulated on having succeeded in a very difficult task, namely that of writing a concise, clear and really practical text-book on the subject of nephritis. This is a book which, although ostensibly written for the student and practitioner, will be constantly referred to by all branches of the profession, and on all points it will be found full of useful information, and clear of the mass of ancient theory and practice which has so obscured our knowledge in the past. No doubt Dr. Alport's classification of the various forms of nephritis will be challenged, but it has the merit of being of real practical service. The various tests of renal efficiency are given in detail, and it is demonstrated that they are such as can be performed by the general practitioner. The relationship between such tests and modern views of the physiology and morbid anatomy of the kidney are clearly set forth, and much emphasis is laid on the important part played by focal sepsis in the causation of nephritis. The question of treatment is most ably handled, and the reasons for using different methods of treatment in each variety of nephritis are explained. There is a valuable appendix of prescriptions for use in renal diseases, and an excellent modern bibliography. The book will do much to establish the diagnosis and treatment of nephritis on a truly scientific basis.

**Autolycus, or the future for Miscreant Youth.** By R. G. GORDON, M.D., D.Sc., F.R.C.P. Pp. 94. London: Kegan Paul, Trench, Trübner & Co. Ltd. 1928. Price 2s. 6d.—An excellent little book, written in a light and easy style, which should be read by parents and schoolmasters, in fact by everyone who is directly interested in the training of children. The carrying out of Dr. Gordon's "aims of the future" will cost money, but to deal in any way seriously with the problem of mental deficiency money must be spent. Although only 8 per cent. of the miscreant youth themselves can be certified under the Mental Deficiency Acts, surely at least one of their progenitors is, in most cases, mentally defective, and when these are dealt with effectively, the time will come when, as Dr. Gordon foresees, "no misfits or defective personalities will be brought into the world." In the Mental Deficiency Act, 1927, the fourth class of defectives are called "moral defectives," but on page 60 the term "moral imbecile" is used. Doubtless this error will be corrected in future editions.

**Elementary Medicine in Terms of Physiology.** By D. W. CARMALT JONES, M.D., F.R.C.P. Pp. viii., 360. 4 figures. London: H. K. Lewis & Co. Ltd. 1929. Price 12s. 6d.—We hope that this book will be widely used by medical students in this country. It has many virtues to commend it. Its author has had, both in England and in New Zealand, a wide clinical experience. He has also been occupied in the teaching of medicine for many years. He has the gift of relating details to principles, of discerning in symptoms and physical signs the aberrations of physiological process. Last, but not least, he has the faculty of expressing very clearly these relations between the normal and the abnormal. His book could be read by any student in a fortnight, and should provide an excellent introduction to clinical medicine.

**The Troubled Conscience.** By C. BLONDEL. Pp. 91. London: Kegan Paul, Trench, Trübner & Co. Ltd. 1928. Price 2s. 6d.—As far as can be gathered from this little book by Charles Blondel, his idea is that cinesthetic (ought it not to be cinæsthetic ?) impulses not ordinarily noticed rise into consciousness and cause feelings of suspicion and fear. The inability to express these or describe them in ordinary language, in spite of our attempts to do so, results in that incoherence of speech and conduct so common in the insane. Unfortunately for this theory, the very cases, viz. hypochondriacal, in which we infer that afferent impulses from the viscera are the cause of the morbid sensations, are not particularly incoherent in speech or violent in conduct. The "poor lunatic" has at any rate justified his existence by supplying material for the numerous theories of dreamers, and the little book is quite worth reading, if only to see what the "reputed sane" will say about the "alleged insane."

**Lipiodol in the Diagnosis of Thoracic Disease.** By F. G. CHANDLER, M.D., F.R.C.P., and W. BURTON WOOD, M.D., M.R.C.P. Pp. viii., 133. Illustrated. London: Oxford University Press. 1928. Price 10s. 6d.—This book, in a short space, gives the present position of knowledge and practice as to the use of lipiodol in pulmonary disease. There is a very useful chapter on the detailed technique for the introduction of lipiodol into the air passages. The actual clinical value of the method is very fairly reviewed and offers much food for thought. The greater part of this small book is taken up with a series of excellent radiograms, which are in themselves a valuable contribution to the literature of the subject.

**The Art of Surgery.** By H. S. SOUTTAR, D.M., M.Ch., F.R.C.S. Pp. viii., 624. Illustrated. London: William Heinemann Ltd. 1929. Price 30s.—This book is of an unusual character, both in the selection of the subjects described and in the method of illustration. The professed ideals at which the volume aims have our most cordial sympathy. The chief of these ideals is that of lightening the student's burden by omitting what is not essential and by describing very fully what is fundamental. But we must confess at the outset that we are disappointed that in both aspects the ideal has not been fully carried out. In the first place we ask ourselves how much of the ordinary text-book contents has been omitted, and as we look through the titles of the chapters we seem to find almost exactly the same subjects as those with which other text-books have made us familiar. Really the only real omission which tends to lighten the student's task is that of operative details and appliances. On the other hand, in spite of the fact that the book contains over 600 pages, it has not been possible to give detailed description of fundamental points. For example, fractures of the femur occupy only three and a half pages. However, in spite of its faults and shortcomings, the book has a great and attractive charm, namely that it represents the opinions and practice of its author, a surgeon of very wide experience and great originality. It is written in a very easy style, and the wide margin containing both headings of paragraphs and thumb-nail sketches make it very easy for reference. It would serve no useful purpose to go through the book, commenting on various opinions about surgical practice, with which we may differ from the author. In regard to the general surgical diseases and principles, the author does not depart widely from generally accepted teaching. In regard to the problems of fractures, we regard the opinions expressed as being rather behind the times. Pott's fracture treated by laying the leg on its outer side on a pillow, fractures of the neck of the femur treated by traction in bed and the omission of reference to non-union and mal-union are examples of this. The sections on cranial surgery are, as one might expect, rather more advanced than is usual in a text-book of this kind. Lastly, we feel that no review would be adequate that did not refer to the remarkable character of the illustrations. Quite frankly we do not like them, but perhaps this is our want of appreciation of modern artistic methods. The coloured illustrations are all of an impressionist character, which may have great merits as works of art, but convey a very poor idea of accurate detail. The thumb-nail sketches vary very much in quality; those which are merely diagrammatic, such as representation of a fracture or dislocation, are very useful, but those which represent the face, for example, in goitres are very poor. No doubt, however, the reputation of the author, the charm of the writing, and the originality of the work will make it very popular in spite of superficial faults, which we hope will be corrected in future editions.

**Elements of Surgical Diagnosis.** By Sir A. PEARCE GOULD, K.C.V.O., C.B.E., M.S., F.R.C.S. Seventh Edition. By E. PEARCE GOULD, M.Ch., F.R.C.S. Pp. xvi., 730. Twenty-six plates. London : Cassell & Co. Ltd. 1929. Price 12s. 6d.—*Surgical Diagnosis* is one of the operator's best friends, for on its accuracy depends chiefly the degree of success attained by the average surgeon. This book occupies a premier position among English surgical text-books, for it embraces a most complete and well-written description of the whole range of surgical disorders both in trunk and limbs. It is good that the author brings it up to date by frequent revisions, and maintains the high repute of the original work. In this edition the application of the radiography of opaque injections for gall-bladder disease and obstruction of the spinal theca are among the lucid radiograms scattered through the book. Most valuable is the solemn warning on p. 478 that it is the doctor's duty to lose no time in settling with certainty the nature of every tumour of the breast. Though this sounds a sorry comment on medical practice, this warning perhaps calls for printing in heavy type at the end of the chapter, for lives are still lost from its neglect. A few omissions are perhaps regrettable. No reference is found to endothoracic goitre, simple retro-peritoneal tumours or that serious and obscure diagnostic puzzle known as carbuncle of the kidney, which stands notably in need of broadcasting. In addition, does not thorough elucidation of pyorrhœa alveolaris call for the aid of X-rays ? But in truth anything other than commendation seems out of place for this masterly text-book.

**The Injection Treatment of Varicose Veins.** By A. H. DOUTHWAITE, M.D., M.R.C.P. Third Edition. Pp. 51. London : H. K. Lewis & Co. Ltd. 1928. Price 4s.—The success of this small book, which has justified the publication of a third edition, is the best evidence of its usefulness. It represents a careful account of what is now recognized as a simple, safe and useful method. It is based upon an experience of nearly 2,000 cases, and the author therefore considers himself justified in describing the essential procedure as perfectly simple, easy of performance, invariably effective and perfectly safe if employed with reasonable care. He claims that the worst cases of varicose veins are those in which the most brilliant results are obtained. In the new edition the main changes relate to alternative solutions which the author has found useful. In the majority of cases he uses a solution of quinine and urethane, and he now suggests that the following solutions are sometimes useful, namely a mixture of sodium salicylate and sodium chloride, sodium chloride alone or a 66 per cent. of glucose. He does not support the suggestion of ligaturing the internal saphenous vein at the same time that injection is made.

**An Index of Symptomatology.** By several Authors. Edited by H. LETHEBY TIDY, M.D., F.R.C.P. Pp. xii., 710. Illustrated. Bristol: John Wright & Sons Ltd. 1928. Price 42s.—We presume this volume completes the *Index* series which has added to the reputation of the publishers. Although at first sight it might seem to overlap the *Diagnosis* volume, a new edition of which appeared recently, a brief examination of its contents and of the way in which they are arranged will prove that this is not so. The reader who takes down the *Diagnosis* volume will do so in order to discover the possible origins and implications of some symptom which he has observed. This new *Symptomatology* volume, on the other hand, tells him what clinical picture to expect in any given disorder. The book is in encyclopædia form, consisting of brief paragraphs headed by names of diseases and alphabetically arranged. This is backed by a supplementary index of several thousand references, and it is therefore easy to find quickly a clinical description of any disease. We advise readers to look up their subjects first under the encyclopædia heading, and if this fails to show them what they want, to rely on the supplementary index. We have tested the book in this way, and find it difficult to discover any serious omission. The paragraphs have been prepared by twenty-six authors, the largest contributor being the indefatigable editor himself, who is to be congratulated on the successful issue of a difficult and prolonged labour. In his preface he invites criticism. We have only one small question to ask, namely whether scurvy is not "C avitaminosis" rather than "vitaminosis." The pictures are excellent, as is usual in the books published by this firm, who have once more demonstrated their capacity for discovering what the medical profession needs, and meeting that need admirably.

**A Patient's Manual of Diabetes.** By Herbert W. Moxon, B.A., M.R.C.S., L.R.C.P. Pp. xi., 132. London: H. K. Lewis & Co. Ltd. 1929. Price 6s. net.—Dr. Moxon, who is Honorary Physician to the Perth Hospital in Perth, Western Australia, has adapted a course of nursing lectures to the needs of his patients. He says in his preface that he considers it impossible to give to patients "too full and complete a grasp of what manner of disease diabetes really is." This perhaps is true provided the "grasp" can be guaranteed. We are inclined to think, however, that an "account" of a disease by an author and the "grasp" of that account by the patient may not be identical. Frankly, Dr. Moxon's physiological descriptions seem far above the understanding of most English patients. For his principles of treatment he relies on such well-established authorities as Maclean, Lawrence and Harrison. He still advocates some degree of preliminary starvation, a method almost entirely abandoned elsewhere and not unattended by danger. He is not quite convinced that insulin has made pregnancy safe for the diabetic woman. The book is well printed and well arranged.

**Maternity and Child Welfare.** By Ethel Cassie, M.D., Ch.B., D.P.H. Pp. viii., 228. Illustrated. London: H. K. Lewis & Co. Ltd. 1929. Price 8s. 6d.—Dr. Cassie's book on maternity and child welfare is written as the result of excellent practical experience, and should be read by all nurses who are thinking of taking up health visiting as a profession. It may also be read with advantage by those to whom this work is little more than a name; to them it will give a good general impression of the valuable work undertaken by this department. Although Dr. Cassie mentions the fundamental principle of child welfare work, and points out that for the purely clinical nurse it has no real appeal, more stress might have been laid in the opening chapters on the fact that the work is essentially preventive; that the aim, in addition to the instruction of the ignorant mother, is the supervision of the healthy child, and the handing over to medical care of the mother or child as soon as the earliest signs of disease appear. Some of the chapters in Sections 2 and 3 appear to be somewhat out of place, as, of necessity, they are not detailed enough to be really instructive to the trained worker; but the health visitor, who is beginning her work, will find much that is useful in the book, particularly the chapters on infant feeding and child management, and the schedule which gives an outline of the most important points to be dealt with in home visiting.

**Deafness and its Alleviation.** By V. NESFIELD, F.R.C.S. Pp. vi., 85. 19 illustrations. London: H. K. Lewis & Co. Ltd. 1928. Price 7s. 6d.—In the earlier pages of this work a brief but lucid account of the anatomy and physiology of the ear is provided, followed by a description of the nature of deafness, including that due to acute and chronic suppuration. Then treatment is considered, including that of the related conditions of the nose and throat. Many useful hints for the general management of the deaf are given in this section. Later the author writes: "But if the patient is more than a little deaf, and particularly if incapacitated by deafness, then I advise operation." The operation consists in making an opening into the attic of the tympanum through the mastoid process, close behind the osseous meatus. Part of the latter is removed and a meatal flap turned back. An extra "pocket" is "given to the middle ear . . often quite sufficient to keep the Eustachian tube patent, by giving an additional 1 c.c.m. expanding space." The author has operated on over 300 patients in this way. A few case records are appended. In some of these the improvement of hearing power recorded, even when apparent nerve-deafness existed previously, was little short of miraculous. The book is one which will repay perusal. The earlier portion will be helpful to the general practitioner, and the sections on the operative treatment cannot fail to interest specialists and challenge their criticism.

**Principles of Clinical Pathology in Practice.** By G. BOURNE, M.D., M.R.C.P., and K. STONE, M.D., M.R.C.P. Pp. xii., 392. Illustrated. London: Oxford University Press. 1929. Price 16s.—This book deals principally with the theoretical side of clinical pathology. The intention of the authors is to supply the clinician with sufficient information to decide the necessary tests and interpret the laboratory findings. The technical details of the various tests have been omitted, except where their inclusion would help towards a proper understanding of the pathology of the disease in question. There are eight sections, which deal in turn with specific infections, diseases of the blood, diseases of the cardiovascular, gastro-intestinal, respiratory and urogenital systems and metabolic and allergic diseases. The pathology of each disease is described, the methods of investigation are indicated and the correct interpretation of the test findings explained. An appendix contains directions for the collection and preservation of material. There is an extensive list of references at the end of each section. The book is particularly suited to the clinician's needs, and we can confidently recommend it.

**Practical Chiropody.** Third edition. By E. G. V. RUNTING. Pp. xii., 200. London: Faber & Gwyer Ltd. 1928. Price 5s.—In these days of ready-made and often badly designed footwear a very large proportion of our population is more or less severely crippled by corns, bunions and other painful affections of the feet. The busy general practitioner has often insufficient time to treat these conditions adequately, and is very glad to hand them over to the chiropodist. It is for the latter that this book is written. The etiology, pathology and treatment of minor, but none the less important, foot troubles are clearly and simply set out. Mr. Runting's book should prove of great value to the chiropodist. We are glad to notice that stress is laid upon the importance of referring to a qualified medical man all those cases which do not yield to treatment, or which do not fall strictly within the sphere of the chiropodist. The practitioner himself might gain several useful hints from a perusal of this handy little book.

**An Index of Differential Diagnosis of Main Symptoms.** By VARIOUS WRITERS. Edited by HERBERT FRENCH, C.B.E., M.A., M.D., F.R.C.P. Fourth Edition. Pp. x:, 1,171. Bristol: John Wright & Sons Ltd. 1928. Price 63s.—The fourth edition of this well-known book will certainly meet with the same welcome as its predecessors. The high standard of the former editions is fully maintained. We have praised the work before, and as time goes on its usefulness, particularly to general practitioners, becomes more widely appreciated. The numerous illustrations add greatly to its value, they have been chosen with excellent judgment and are reproduced (especially the coloured ones) most admirably. The index, extending to 227 pages of small type, is a marvel of completeness and correctness. No section will give a better idea of the scope of the work than the numerous articles dealing with "Pain" (pp. 524–594). Here are thoughtful and suggestive monographs by various authors dealing with regional pain. In another place twenty-four pages are devoted to the causation and recognition of causes of prolonged pyrexia. X-ray diagnosis is among one of the many recent advances which are helpfully discussed. It is a big undertaking to attempt a differential index of symptoms, but Dr. French is to be congratulated on bringing out this new edition and having brought it so admirably up to date. The publishers (Wright of Bristol) maintain easily their position as leaders in illustrated medical literature with this volume.

**Deformities in Infancy and Early Childhood.** By M. F. FORRESTER-BROWN, M.S., M.D. Pp. xii., 199. Illustrated. London: Oxford University Press. 1929. Price 14s.—This book has been written especially for those in charge of obstetrical and infant-welfare clinics, in the hope that it may help them to recognize deformities in infancy and childhood, and, by instituting treatment at the earliest opportunity, may avoid the necessity for those drastic measures which so often become unavoidable in neglected cases. With this aim in view details of treatment are restricted to non-operative procedures. The book is divided into six sections dealing with different regions of the body and limbs. Illustrations have been carefully selected to help out the text, and they are admirably reproduced. We would draw special attention to the educative methods recommended for the cure of postural defects. They are based upon thoroughly sound principles, and should prove of great service. The early treatment of talipes is another subject of great importance which is ably dealt with. Dr. Forrester-Brown has succeeded in compressing a great deal of information into a remarkably small space. We would suggest that the book would in no way suffer were those portions eliminated which refer to uncommon diseases and the remainder served up in a slightly less concentrated form. With this one criticism we would heartily recommend the book to all those interested in the treatment of children. It is a notable addition to the series of Oxford Medical Publications.

**The Tonsils and Adenoids.** By IRWIN MOORE, M.B., C.M. Pp. xx., 395. Illustrated. London: William Heinemann Ltd. 1928. Price 21s.—In his preface the author states that his book embodies a number of papers that he has contributed during recent years on the subject of enlarged tonsils and adenoids, the operative technique for their removal, and the control of hemorrhage during and after removal. On these important subjects the work is a mine of information preceded by a useful introduction on the regional and structural anatomy of the tonsils and their functions and the bacteriology of tonsillar affections, followed by a chapter of much value on the tonsils as a sourse of focal infection. The frequent lack of continuity in the text is doubtless due to incomplete co-ordination of these various papers, which also accounts for overlapping and covering the same ground twice over, *e.g.* contra-indications for removal of tonsils on pp. 296, 297 and 298 are largely a repetition of pp. 142 to 149, and to some extent of pp. 241, 242 and 243. Twice Hugh Ashby is cited on the lymphocyte-forming function of the tonsils, p. 16 and 314. More space should have been accorded to differential diagnosis, *e.g.* between acute tonsillitis and diphtheria, tertiary syphilis and malignant growths, and as only four pages of type are devoted to the entire subject of malignant neoplasms, it would have been better to omit their treatment by diathermy, radium, X-rays or surgical operation, etc., the descriptions of which are so sketchy and very incomplete as to be of little value. The section on the choice of anæsthesia is somewhat disappointing on the question of local anæsthesia now favoured by many operators for tonsil operations on the adult. As an alternative to surface swabbing with cocaine the author suggests "hypodermic injection of 4 per cent. cocaine in a 1 to 2,000 solution of adrenalin, for preference a solution of 2½ per cent. novocaine and 5 per cent. adrenalin," yet very few authorities nowadays resort to cocaine injections at all, and a much less weaker novocaine suffices, and is safe. The author is an enthusiastic convert to the open method of ether anæsthesia, with which we here have been so familiar since its introduction in Bristol towards the close of the last century: Dr. Felix Rood will doubtless be surprised that he is given the credit of introducing open-ether anæsthesia "to this country." The detailed description of the operative technique for enucleation of the tonsils by the guillotine and by dissection, and the instructions for preventing hemorrhage and for arresting it during or after operation are altogether excellent, and enriched with many beautiful illustrations, clear, instructive and helpful, which here, as throughout the book, leave nothing to be desired. These sections and the earlier chapters alone make this monograph of the utmost value to the practitioner.

